# Epidemiology of Penetrating Chest Injuries Presenting at a Tertiary Care Center in Peshawar: A Retrospective Study

**DOI:** 10.7759/cureus.65987

**Published:** 2024-08-02

**Authors:** Abdul Baseer, Pralaya Khadka, Yasir Badshah, Muhammad Hammad Khan

**Affiliations:** 1 Cardiothoracic Surgery, Medical Teaching Institute, Lady Reading Hospital, Peshawar, PAK; 2 Thoracic Surgery/Cardiothoracic Surgery, Medical Teaching Institute, Lady Reading Hospital, Peshawar, PAK

**Keywords:** injury, hemothorax, pain and discomfort, patients, chest discomfort

## Abstract

Introduction: Trauma is the leading cause of mortality globally. Penetrating chest trauma is a type of injury that occurs when an object pierces the skin and enters the chest wall. The incidence of penetrating chest trauma is high in the Khyber Pakhtunkhwa Province of Pakistan, with firearm injuries being the most common cause.

Objective: The objective of this study is to determine the epidemiology and outcome of non-cardiac penetrating chest injuries presented at a tertiary care center in Peshawar.

Material and methods: This retrospective cohort study was conducted from January 2022 to January 2023 at Medical Teaching Institute, Lady Reading Hospital, Peshawar, KPK, Pakistan. Two hundred and three patients who suffered penetrating chest trauma in the Khyber Province of Pakistan between January 2022 and January 2023 were included in this retrospective cohort study. The epidemiology and outcomes were analyzed using IBM SPSS Statistics for Windows, Version 23 (Released 2015; IBM Corp., Armonk, New York, United States).

Results: The mean age of the patients was 30.25 ± 16.674 years. Males comprised 183 (90.1%) of the study sample whereas females comprised only one-tenth 20 (9.9%). Gunshot injuries were the predominant mode of penetrating thoracic trauma comprising 128 (63.05%) of all injuries. Injuries inflicted by knife constituted approximately one-fifth of the presentations 44 (21.67%). The remaining injuries labeled ‘Others’ comprised modes such as road traffic accidents and fall injuries where the predominant mechanism was penetrating injury. The latter comprised 31 (15.27%) of the injuries.

Conclusion: Penetrating chest trauma is common in the Khyber Pakhtunkhwa Province of Pakistan. Gunshot injury is the most common mode. Males are predominantly affected. Most non-cardiac penetrating chest trauma can be managed conservatively.

## Introduction

Trauma is the leading cause of mortality worldwide. While blunt chest trauma accounts for about 90% of all chest traumas, penetrating injuries make up one-tenth [1]. One-fourth to one-fifth of mortality in trauma occurs as a result of thoracic injuries [2]. The incidence of penetrating chest trauma can differ based on urban or rural setup. Overall, penetrating chest injuries comprise about 10% of trauma admissions, with approximately 1 in every 10 requiring operative interventions. The number is higher for those unstable at presentation. Among stable patients, about one in every four develops complications that include retained hemothorax, empyema thoracis, prolonged air leak, and occult diaphragmatic injuries [3-6].

Penetrating thoracic trauma can be managed conservatively in most cases i.e. with observation or tube thoracostomy. Close to 15% require operative intervention either via a thoracotomy or video-assisted thoracoscopic surgery for complications like hemothorax [7]. Immediate drainage of at least 1.5 liters of blood or an output of 200 ml every hour for at least three hours prompts immediate exploration and so does unstable hemodynamics regardless of the drainage volume [8].

In Pakistan, penetrating injuries especially due to gunshots and knives pose a substantial health concern [9]. The youth in particular have an alarmingly high usage of weapons with unknown ownership. Approximately one-third of patients with gunshot injuries presenting to our center have injuries to the chest [10-12].

This study aims to present data on the epidemiology and outcomes of non-cardiac penetrating chest trauma presenting at a tertiary care center in the Khyber Province of Pakistan, where the incidence of firearm injuries among other modes of penetrating chest injuries is evident. 

## Materials and methods

This is a retrospective study done at Medical Teaching Hospital, Lady Reading Hospital, Peshawar, KPK, Pakistan between January 2022 and January 2023 for a total of 12 months. The center received a total of 237 cases of penetrating injuries during the period of study.

Inclusion and exclusion criteria

Patients aged > 18 years who visited the hospital were included in the study. Based on the study criteria, 34 patients were excluded. Among them, eight were declared dead on arrival at the emergency department, five had associated cardiac injuries with four of them non-resuscitable and died during exploration, and one revived after exploration in the emergency department, eight had blunt and penetrating trauma with blunt being the major mechanism, nine had diaphragmatic injuries at abdominal exploration, and four had knife injuries without pleural breach. After excluding these patients, the remaining 203 were included in the study.

Data collection

Data were collected according to the inclusion and exclusion criteria of the study. Data were collected retrospectively. All patients sustaining penetrating thoracic injuries were included after initial stabilization in the emergency department and written informed consent was taken from patients’ relatives. All patients who required chest tube insertion were hospitalized. All patients who had an initial drainage of 1500 ml or more of blood were taken to the operating room for emergent thoracotomy. Also, patients with tube drainage of greater than 200 ml of blood every hour for at least three hours, and those with any amount of drain output with unstable vitals were explored.

Data were then entered and analyzed using IBM SPSS Statistics for Windows, Version 23 (Released 2015; IBM Corp., Armonk, New York, United States). All the continuous descriptive variables were represented as mean and SD and all the categorical data were presented as frequency and percentages. Normality of the data was checked on MS Excel.

## Results

Out of two hundred and three patients enrolled in the study, 183 were men and 20 were women. Males comprised 90.1% of the study sample whereas females comprised only one-tenth (9.9%). The mean age of selected patients was 30.25 ± 16.674 years. Table 1 explains that the majority of the patients were residents of Peshawar (32.5%), Charsadda (9.9%) and Swabi (5.4%). Gunshot injuries were the predominant mode of penetrating thoracic trauma comprising 63.05% of all injuries. Injuries inflicted by knives constituted approximately one-fifth of the presentations (21.67%). The remaining injuries labeled ‘Others’ comprised modes such as road traffic accidents and fall injuries where the predominant mechanism was penetrating injury. The latter comprised 15.27% of the injuries (Table 1).

**Table 1:** Demographic and baseline data of injury of participants

Characteristic	Number (n=203)	Percentage (%)
Gender		
Male	183	90.1
Female	20	9.9
Age (years)		
Mean (± SD)	30.25 ± 16.674	
Median	26	
Range	1 - 100	
Residency		
Peshawar	66	32.5
Charsadda	20	9.9
Swabi	11	5.4
Other	106	52.2
Mode of Penetrating Thoracic Trauma		
Gunshot	128	63.05
Knife	44	21.67
Others (e.g., Road Traffic Accidents, Fall Injuries)	31	15.27

Table 2 showed that the predominant site of entry was the supramammary region of the thorax with 115 (56.7%) patients sustaining injury over the area. The next most common sites were the inframammary (21.7%) and the infrascapular (10.3%) regions. Other entry sites included infra-axillary (6.4%) and interscapular (4.9%). There were no exit wounds in 82 patients (40.4%). Supramammary exits were predominant (21.2%) followed by interscapular (12.8%) and infrascapular (11.8%). Other exits sites included inframammary (9.4%) and infra-axillary (4.4%). Eleven patients of the total cohort also sustained spine injuries with or without paraplegia, eight of whom had gunshot injuries. Two patients with knife injury and one from other penetrating mechanisms suffered concomitant injury to the spine (Table 2).

**Table 2:** Injury characteristics and study outcomes

Characteristic	Number (n)	Percentage (%)
Gender Distribution		
Gunshot Injuries (n=128)		
Male	115	89.8
Female	13	10.2
Knife Injuries (n=44)		
Male	42	95.5
Female	2	4.5
Other Injuries (n=31)		
Male	26	83.9
Female	5	16.1
Side of Injury		
Left Hemithorax	123	60.6
Right Hemithorax	59	29.1
Bilateral	21	10.3
Entry Wounds		
Single-Entry Wound	163	80.3
Multiple-Entry Wounds	40	19.7
Exit Wounds		
Single-Exit Wound	101	49.8
Multiple-Exit Wounds	20	9.9
No Exit Wounds	82	40.4

According to the analysis shown in Table 3, 172 (84.7%) of the patients could be managed with a chest tube alone, and 31 (15.3%) required exploration. All thoracotomies were done for either an immediate drain output of greater than 1.5 liters or hourly drainage in excess of 200 ml for at least three hours. All explorations were done via standard posterolateral thoracotomy. Most of the thoracotomies were needed for gunshot injuries. Twenty-seven out of 31 of the thoracotomies were done for gunshot, one for knife injury and three for penetrating injuries from other mechanisms. ICU admission was done for patients with hemothorax with immediate drain output volume between 500 and 1500 ml, and for patients who required thoracotomy for control of intrathoracic hemorrhage. Altogether 50 patients (24.6%) required admission in the ICU (Table 3).

**Table 3:** Injury entry and exit site with management of wound

Characteristic	Number (n)	Percentage (%)
Site of Entry Wounds		
Supramammary	115	56.7
Inframammary	44	21.7
Infrascapular	21	10.3
Infra-axillary	13	6.4
Interscapular	10	4.9
Site of Exit Wounds		
None	82	40.4
Supramammary	43	21.2
Interscapular	26	12.8
Infrascapular	24	11.8
Inframammary	19	9.4
Infra-axillary	9	4.4
Spine Injuries		
Total	11	5.4
Gunshot	8	72.7
Knife	2	18.2
Other	1	9.1
Management		
Chest Tube Alone	172	84.7
Exploration (Thoracotomy)	31	15.3
Thoracotomy Indications		
Gunshot	27	87.1
Knife	1	3.2
Other	3	9.7
Findings at Thoracotomy		
Intercostal Bleeding	13	41.9
Lung Laceration	14	45.2
Both Intercostal Bleeding and Lung Laceration	2	6.5
Lung Contusion without Active Bleeding	1	3.2
Scapular Fracture	1	3.2
Outcomes		
Hemothorax	189	93.1
Pneumothorax	118	58.1
Rib Fractures (Single/Multiple)	27	13.3
ICU Admissions	50	24.6

## Discussion

Penetrating chest wound patients usually reach the hospital alive. A thorough head-to-toe examination is essential in every case with particular attention to the trajectory taken by the penetrating object [13]. Initial assessment should begin with airway and volume status and prompt management accordingly. Out of the 128 patients afflicted by gunshot injuries, males were predominant (115/128; 89.8%), whereas only 13 (10.2%) were females. A total of 44 patients sustained knife injuries Again, almost all of the injured were men. Forty-two males in comparison to only two females were afflicted by stab injuries with knives. Mechanisms of injury other than gunshot or knife included fall and road traffic accidents with penetrating injury being the major mechanism. Here again, male predominance was detected as 26 of 31 cases were males and only five were females. Overall, there were eight mortalities (3.94%). Six out of 128 gunshot injuries (4.6%) resulted in deaths, whereas no deaths occurred in those with knife injuries. Mechanisms other than gunshot and knife injuries resulted in two mortalities (6.4%) The predominant side of injury was the left hemithorax with 123 cases presenting with left-sided injuries. The right hemithorax was injured in 59 patients. Bilateral injuries occurred in 21 cases. Regarding entry wounds, most patients (163/203) had single-entry wounds, whereas 40 patients had multiple-entry wounds. Twenty patients had multiple-exit wounds, whereas 101 patients had single-exit wounds. Eighty-two patients had no exit wounds [14]. The objectives of treatment in penetrating injuries are the evaluation of hemothorax, pneumothorax, and/or hemopneumothorax. A proper physical examination and chest x-ray provide valuable initial information on the need for a tube thoracostomy. For a large effusion, physical examination has a sensitivity of 96% and a specificity of 93% respectively. However, a normal examination does not rule out pleural pathology. Physical examination is normal in up to 28% of patients with small pneumothorax. Penetrating chest injury is more common among the younger population. Our study showed that the age group falling in the 20 to 30 years of age suffered most of the trauma. Similarly, 96% of firearm injury victims were males in the study by Shah et al. [15]. Firearm injuries are also the leading cause of death among African American males between the ages of 15 and 34 years. Male predominance is stark in Pakistan, Turkey, and Saudi Arabia. It is narrower in western nations like Greece [16].

A major proportion of homicides are caused by firearm injuries [17]. Domestic violence was found to be the most common reason for firearm injuries in a study by Afridi et al. (44%) [12]. The firearms commonly used in inflicting gunshot wounds are pistols (70%), snipers (9%), shotguns, and other weapons (5%). The same study found that firearm injuries presented to Lady Reading Hospital Peshawar were 72% homicidal and 14% suicidal. Isolated chest injuries were the third most common area of gunshot injuries after lower limbs and abdominal-pelvic [18]. In our study of 203 patients, there were altogether eight mortalities (3.9%). Their mean age was 33.25 years. Three-fourths of these mortalities resulted from gunshot wounds. In a study by Marri et al., it was observed that 96% of mortalities from firearm injuries occur due to multiple and 4% from single bullet injuries [19]. Their study of 100 homicidal deaths due to rifled weapons presenting for autopsy revealed that the chest was the most common site of injury (33.8%) in those where the motive was to kill and limbs the common site in cases where the motive was only to injure. One-fifth (19.7%) had multiple entry wounds. The mortality rate among those with multiple-entry wounds in comparison to those with single-entry wounds was statistically significant (p=0.028). This suggests that those with multiple-entry wounds were significantly more likely to die than those with single-entry wounds. The major findings at thoracotomy were intercostal bleeding in 13 patients and lung laceration in 14 patients with both present in two patients. Besides that, lung contusion without active bleeding from the lung was seen in one patient, and an associated scapular fracture in another. Hemothorax was the most common result of penetrating thoracic injuries comprising 93.1% of the cases. This was followed by pneumothorax which was seen in 118 patients (58.1%). Rib fractures, single and multiple, occurred in 27 cases (13.3%).

The majority of entry wounds were on the left hemithorax. This may be explained by the predominance of right-handed people in the general population, close to 90%. About three-fourths of entry wounds occurred in the supramammary region and inframammary regions which reflects that most of the assaults occur in a face-to-face manner, which would also explain the left-sided injuries in most of the victims inflicted by mostly right-handed assaulters. In our study, 4.6% of patients died from gunshot injuries. This is comparable to the study by Shah et al. in which 6.8% of the study cohort succumbed as a result of firearm injuries. Considering all eight mortalities, six were males indicating that males are three times more likely than females to die from a penetrating chest wound reiterating the fact that males are predominantly at risk of penetrating injury and mortalities thereof. Trans-mediastinal and transabdominal injuries associated with penetrating chest trauma increase mortality substantially. Two studies have reported mortalities of 36-42% in such scenarios. Hence, these were not included in our study.

Limitations of the study

This study provides valuable insight into penetrating chest trauma in a region of KPK, Pakistan. This study also provides some management strategies to overcome penetrating chest trauma. This study guides about management and infection control after injury happening with multiple reasons but there are several limitations of the study which are also present. This is a single-center study conducted in Peshawar so we may not generalize this study to the whole population. Data is biased and non-random sampling was utilized. The primary focus of the study is on penetrating chest trauma without long-term follow-up. So, there is no follow-up for patients. Differences in clinical management and approaches provided by doctors may vary according to area and it may also change the outcomes of the study. This study also excludes non-penetrating injuries.

## Conclusions

Penetrating chest trauma is common in Pakistan, especially Peshawar region. Gunshot injuries make up most of the injuries followed by knife injuries and males are the predominant victims. Most of the non-cardiac penetrating chest injuries can be managed conservatively and the prognosis is favorable.
